# Validation of CLIF-C ACLF score to define a threshold for futility of intensive care support for patients with acute-on-chronic liver failure

**DOI:** 10.1186/s13054-018-2156-0

**Published:** 2018-10-10

**Authors:** Cornelius Engelmann, Karen Louise Thomsen, Nekisa Zakeri, Mohammed Sheikh, Banwari Agarwal, Rajiv Jalan, Rajeshwar P. Mookerjee

**Affiliations:** 1 Institute for Liver and Digestive Health, University College London, Royal Free Campus, Rowland Hill Street, London, NW32PF UK; 2 Section of Hepatology, Department of Internal Medicine, Neurology, Dermatology, University Hospital Leipzig, Liebigstrasse 20, 04103 Leipzig, Germany; 3 Department of Hepatology and Gastroenterology, Aarhus University Hospital, Nørrebrogade 44, 8000 Aarhus C, Denmark; 4 Intensive Care Medicine, Royal Free Hospital, Pond Street, London, NW32QG UK

**Keywords:** ACLF, Futility, Cirrhosis, Intensive care unit

## Abstract

**Background:**

Acute-on-chronic liver failure (ACLF) is a severe complication of cirrhosis and is defined by organ failure and high rates of short-term mortality. Patients with ACLF are managed with multiorgan support in the intensive care unit (ICU). Currently, it is unclear when this supportive care becomes futile, particularly in patients who are not candidates for liver transplant. The aim of this study was to determine whether the currently available prognostic scores can identify patients with ACLF in whom prolonged ICU care is likely to be futile despite maximal treatment efforts.

**Methods:**

Data of 202 consecutive patients with ACLF admitted to the ICU at the Royal Free Hospital London between 2005 and 2012 were retrospectively analyzed. Prognostic scores for chronic liver diseases, such as Child-Pugh, Model for End-Stage Liver Disease (MELD), European Foundation for the study of chronic liver failure (CLIF-C) organ failure (OF), and CLIF-C ACLF, were calculated 48 hours after ICU admission and correlated with patient outcome after 28 days.

**Results:**

The CLIF-C ACLF score, compared with all other scores, most accurately predicted 28-day mortality, with an area under the receiver operator characteristic of 0.8 (CLIF-C OF, 0.75; MELD, 0.68; Child-Pugh, 0.66). A CLIF-C ACLF score cutoff ≥ 70 identified patients with a 100% mortality within 28 days. These patients had elevated inflammatory parameters representing a systemic inflammatory response, most often renal failure, compared with patients below this cutoff.

**Conclusions:**

Patients with ACLF and high CLIF-C ACLF score (≥ 70) after 48 hours of intensive care may reach a threshold of futility for further ongoing intensive support. The best treatment options in this scenario remain to be determined but may include palliative care.

**Electronic supplementary material:**

The online version of this article (10.1186/s13054-018-2156-0) contains supplementary material, which is available to authorized users.

## Background

Acute-on-chronic liver failure (ACLF) is a syndrome that develops in patients with an acute decompensation of liver cirrhosis and is characterized by development of organ failure and high short-term mortality [1]. The diagnostic criteria for organ failure and subsequent ACLF gradation are based on the European Foundation for the study of chronic liver failure (CLIF) organ failure score (CLIF-OF score), a modified version of the Sequential Organ Failure Assessment (SOFA) score [1, 2]. Depending on the ACLF grade, 28-day mortality ranges from 23.3% in ACLF grade 1 to 75.5% in ACLF grade 3 [1], and most patients require intensive care and organ support [3, 4].

In order to prognosticate mortality in patients with ACLF more accurately, the CLIF consortium derived and validated a new score, the CLIF-C ACLF score [2]. The CLIF-C ACLF score combines CLIF-OF score with patients’ age and white blood cell (WBC) count to generate a composite score of 0–100 in a linear range. Validation in an external prospective cohort showed that this score was significantly more accurate than Child-Pugh score, Model for End-Stage Liver Disease (MELD) score, and MELD with serum sodium score in predicting 28-day mortality in ACLF [2]. CLIF-C ACLF score predicted short-term mortality 25% better than all listed scores [2]. The 28-day mortality varied from below 20% in CLIF-C ACLF score < 45 to more than 80% in CLIF-C ACLF score ≥ 65 [2].

The utility of CLIF-C ACLF score in patients with ACLF grade 3, and specifically CLIF-C ACLF score > 64, has been discussed [5, 6] because these patients may still have a poor prognosis in spite of maximal treatment efforts and the associated high costs. Validating the CLIF-C ACLF score on the dataset of the CANONIC (EASL-CLIF Acute-on-Chronic Liver Failure in Cirrhosis) study has shown that in a subset of patients with four or more organ failures and/or CLIF-C ACLF score ≥ 65, 3–7 days after ACLF diagnosis, mortality rates were 100%. Single-center experiences in a small subset of such patients with ACLF (*n* = 23) presented by Cardoso et al. [6] supported this notion, albeit that mortality in this cohort was lower at 86% after 90 days [5]. As a consequence, it has been suggested that intensive care support could be withdrawn in patients with this severity of disease. However, because the available data to support this notion are restricted to the CANONIC cohort and one small, single-center, study, further validation is required before this can be considered for translation into clinical practice.

In this study, we aimed to evaluate the short-term outcome of patients with ACLF and compared the predictive value of the CLIF-C ACLF score against other prognostic scores and clinical variables 48 hours after full intensive care support and regardless of when ACLF was first diagnosed. We also aimed to determine whether the CLIF-C ACLF score could be used to define the futility of ongoing intensive care unit (ICU) support.

## Methods

### Patients and study design

In this retrospective single-center study, data of 202 consecutive patients with ACLF admitted to the ICU at the Royal Free Hospital London were analyzed. All patients received organ system support, including mechanical ventilation, renal replacement therapy, and vasopressor support as required. All parameters at 48 hours of ICU admission were used to diagnose ACLF and to calculate prognostic scores. The parameters included demographic and biological variables and the number of organs that failed. Data for this study were obtained through archived patient notes, collected between 2005 and 2012 in the hospital, and the follow-up data 28 days after ICU admission were retrieved through a combination of the follow-up clinic notes, patients’ general physicians, and direct telephone contact with patients themselves. This database is updated at regular intervals, and some of the patients have previously been analyzed to determine predisposing factors leading to ACLF [7] for use as the validation cohort for the CLIF-C ACLF study [2] and to clarify the role of ammonia, inflammation, and oxygenation in brain dysfunction in ACLF [8].

### Diagnostic criteria for ACLF and management

Criteria for the diagnosis of ACLF was made using the CLIF-OF classification, which is a modification of the CLIF-C SOFA score [1, 2]. Organ failures were defined as follows according to the method of Moreau et al. [1]: renal failure as serum creatinine ≥ 2 mg/dl and/or requirement for renal replacement therapy; brain failure as hepatic encephalopathy graded III/IV according to the West Haven Criteria; liver failure, defined as bilirubin ≥ 12 mg/dl; coagulation failure as international normalized ratio (INR) ≥ 2.5; circulation failure, defined as treatment with vasoconstrictors to maintain the arterial blood pressure or to increase the cardiac output; and lung failure as a partial pressure of oxygen/fraction of inspired oxygen ratio ≤ 200 or peripheral capillary oxygen saturation/fraction of inspired oxygen ratio ≤ 214. ACLF grade 1 was defined by the presence of single kidney failure or any other organ failure when in combination with either renal insufficiency (serum creatinine ≥ 1.5 mg/dl) or hepatic encephalopathy grade 1/2. The ACLF grade 2 or 3 was defined by the presence of two or at least three organ failures, respectively.

### Prognostic score calculation

The CLIF-C ACLF score was calculated by combining the CLIF-C OF score, age, and WBC count with the following formula: CLIF-C ACLF = 10 × (0.33 × CLIF-OFs + 0.04 x Age + 0.63 × ln(WBC count) − 2 [2]. The MELD score and Child-Pugh score were calculated as described previously [9]. The systemic inflammatory response syndrome (SIRS) score expressed the number of SIRS criteria components that were fulfilled.

### Statistics

Variables were tested for a normal distribution using quantile-quantile plots and histograms. Differences in normally distributed continuous variables were evaluated by Student’s *t* test, whereas variables showing skewed distributions with variance heterogeneity were evaluated by the Mann-Whitney *U* test. Pearson χ^2^ test was used to compare categorical variables. The accuracy of the CLIF-C ACLF score in predicting survival was assessed by calculating the area under the receiver operating characteristic (AUROC) curve. A cutoff value was chosen to accurately predict fatalities with a high specificity. Survival analysis was performed according to the CLIF-ACLF cutoff values by using Kaplan-Meier analysis and log-rank test for group comparison. Because only one patient was transplanted in the whole cohort, this event was not considered to be a competing risk. Univariate analysis was carried out to identify the baseline factors associated with occurrence of death (*see* Additional file 1). A multivariate Cox regression model was then fitted individually for each prognostic score with identified potentially confounders of death (*p* < 0.2) in this cohort. All potential confounders that were part of predictive score calculations (MELD, Child-Pugh, CLIF-C ACLF) were not included in the multivariate model. Patients lost to follow-up were censored at the time of last patient contact. Normally distributed data are presented as mean ± SD, and nonparametric data are presented as median (IQR). A two sided *p* value < 0.05 was considered statistically significant.

## Results

### Clinical characteristics of patients

Of the 202 patients included in the study, 99 died within 28 days and 1 was transplanted. In relation to deaths within 48 hours, of the 202 patients included in the study, 9 ACLF grade 3 and 6 ACLF grades 1 + 2 patients died just within this time point, albeit that their retrospective ACLF score classifications were based on the last available data points premortem.

**Fig. 1:**
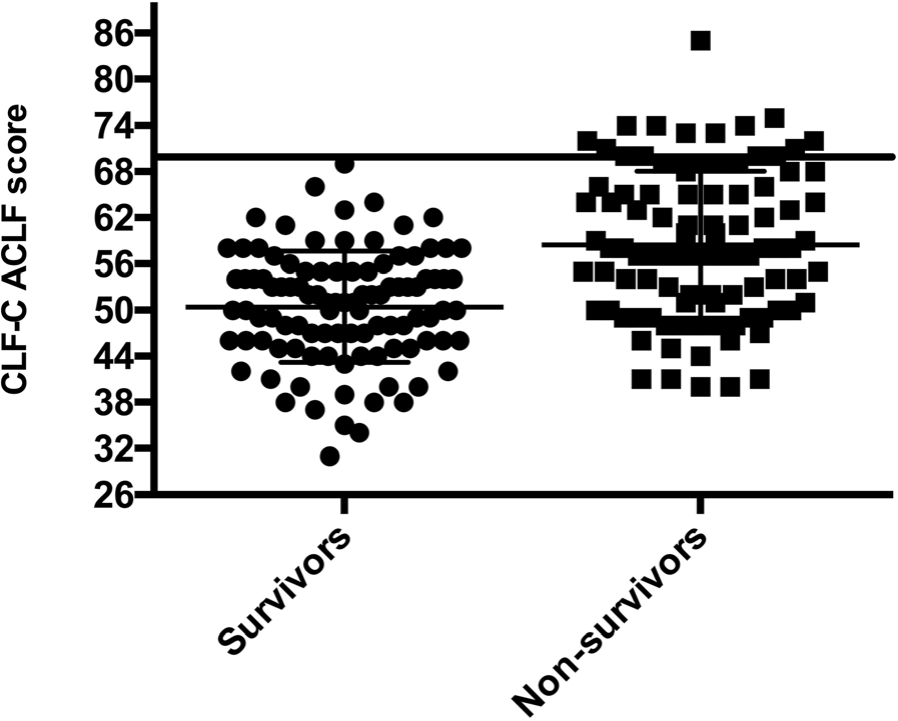
Individual European Foundation for the study of chronic liver failure (CLIF-C) Acute-on-Chronic Liver Failure (ACLF) scores of survivors and nonsurvivors. The bar represents the CLIF-C ACLF score threshold above which futility of care should be considered

Whereas bilirubin levels (3.4 mg/dl vs. 7.8 mg/dl, *p* < 0.001), INR (1.8 vs. 2.2, *p* < 0.0001), and serum creatinine (0.9 mg/dl vs. 1.5 mg/dl, *p* = 0.002) were higher in patients who died, the sodium level, platelet count, albumin level, and WBC count were not different between survivors and nonsurvivors. Of the patients who survived, 26 (25%) were treated with renal replacement therapy, whereas 41 (41%) (*p* = 0.02) of the nonsurvivors received this therapy. The gender distribution, age, and prevalence of hepatic encephalopathy were similar in both groups. Patients who died more often had a higher number of organ failures than the survivors (4–6 organ failures, survivors 2% vs. nonsurvivors 22%, *p* < 0.0001). The same applied to the CLIF-OF score, which was higher in nonsurvivors (median, 13 [11–14] vs. 11 [9–12]; *p* < 0.0001). All prognostic scores, defining the severity of liver dysfunction, were markedly increased in nonsurvivors. MELD score was 30 ± 10 in nonsurvivors compared with 23 ± 9 in survivors (*p* < 0.0001). In total, 92% of nonsurvivors and 71% of survivors (*p* < 0.0001) had Child-Pugh grade C. The CLIF-C ACLF score of 58.4 ± 9.6 was also statistically higher in patients who died, compared with 50.6 ± 7.3 in survivors (*p* < 0.0001) (Table 1, Fig. 1).

**Table 1:** Clinical parameters according to survival status after 28 days

Parameter	Alive (*n* = 103)	Dead (*n* = 99)	*p* Value
Male sex, *n* (%)	70 (68%)	66 (67%)	*p* = 0.85
Age, years	50 ± 12	53 ± 11	*p* = 0.19
MELD score	23 ± 9	30 ± 10	*p* < 0.0001
Child-Pugh score	10.5 ± 1.7	11.9 ± 1.6	*p* < 0.0001
Child-Pugh classification, A/B/C, *n* (%)^a^	2/28/73 (2%/27%/71%)	0/8/90 (0%/8%/92%)	*p* = 0.001
CLIF-OF score	11 (9–12)	13 (11–14)	*p* < 0.0001
CLIF-C ACLF score	50.6 ± 7.3	58.4 ± 9.6	*p* < 0.0001
Number of organ failures, 1–3/4–6, *n* (%)	101/2 (98%/2%)	77/22 (78%/22%)	*p* < 0.0001
Renal replacement, *n* (%)	26 (25%)	41 (41%)	*p* = 0.02
HE classification, 0–2/3–4^b^, *n* (%)	91/12 (88%/12%)	78/21 (79%/21%)	*p* = 0.07
Bilirubin, mg/dl; μmol/L	3.4 (1.6–9.1); 58 (27–156)	7.8 (3.5–15.5); 133 (60–265)	*p* < 0.001
INR	1.8 (1.5–2.3)	2.2 (1.9–3.1)	*p* < 0.0001
Albumin, g/dl; g/L	2.6 ± 0.7; 26 ± 7	2.5 ± 0.7; 25 ± 7	*p* = 0.23
Platelet count, 10^9^/L	87 (57–137)	80 (53–119)	*p* = 0.17
Sodium, mmol/L	138 ± 9	137 ± 10	*p* = 0.20
Serum creatinine, mg/dl; μmol/L	0.9 (0.7–1.6); 80 (62–142)	1.5 (0.9–2.4); 133 (80–212)	*p* = 0.002
WBC count, 10^9^/L	9.4 (6.3–15.1)	10.2 (6.3–16.5)	*p* = 0.37

*Abbreviations: ACLF* Acute-on-Chronic Liver Failure, *CLIF* European Foundation for the study of chronic liver failure, *HE* Hepatic encephalopathy, *INR* International normalized ratio, *MELD* Model for End-Stage Liver Disease, *OF* Organ failure, *WBC* White blood cell

Categorical variables are displayed in percent and continuous variables as mean ± SD (normally distributed data) or median (IQR) (nonparametric testing)

Ascites grades: 0 = no ascites/slight ascites; 1 = moderate ascites; 2 = severe/refractory ascites

^a^No patient in ACLF 3 was allocated to Child-Pugh class A

^b^Classification according to West Haven Criteria [25]

### Predictors of 28-day mortality

All prognostic scores were tested individually with significant confounders in a Cox regression analysis to assess and compare their respective predictive abilities. Univariate analysis was carried out to identify potential predictors of 28-day mortality in a univariate Cox regression model (*see* Additional file 1). Variables that were included in the score calculations were not considered for this analysis. Results disclosed CLIF-C ACLF score (HR = 1.07; 95% CI 1.05–1.09; *p* < 0.0001), the Child-Pugh score (HR = 1.35; 95% CI 1.20–1.53; *p* < 0.0001), and the MELD score (HR = 1.05; 95% CI 1.03–1.07; *p* < 0.0001) as the independent predictors of 28-day mortality (Table 2).

**Table 2:** Multivariate analysis by Cox regression to adjust prognostic scores with confounders associated with 28-day mortality

MELD score			Child-Pugh score			CLIF-C ACLF score		
Parameter	HR	*p* Value	Parameter	HR	*p* Value	Parameter	HR	*p* Value
MELD score	1.05 (95% CI 1.03–1.07)	< 0.0001	Child-Pugh score	1.35 (95% CI 1.20–1.53)	< 0.0001	CLIF-C ACLF score	1.07 (95% CI 1.05–1.09)	< 0.0001
Age (years)	1.01 (95% CI 1.00–1.03)	0.12	Age (years)	1.02 (95% CI 1.00–1.04)	0.04			
Albumin (g/L)	0.98 (95% CI 0.95–1.01)	0.17				Albumin (g/L)	0.99 (95% CI 0.96–1.02)	0.41
Platelet count (10^9^/L)	0.97 (95% CI 0.99–1.00)	0.04	Platelet count (10^9^/L)	1.00 (95% CI 0.99–1.00)	0.07	Platelet count (10^9^/L)	0.99 (95% CI 0.99–1.00)	0.002
HE (0–2/3–4)	1.31 (95% CI 0.80–2.15)	0.29						

*Abbreviations: ACLF* Acute-on-Chronic Liver Failure, *CLIF* European Foundation for the study of chronic liver failure, *HE* Hepatic encephalopathy, *MELD* Model for End-Stage Liver Disease

### Predictors of mortality in ACLF grade 3

**Fig. 2:**
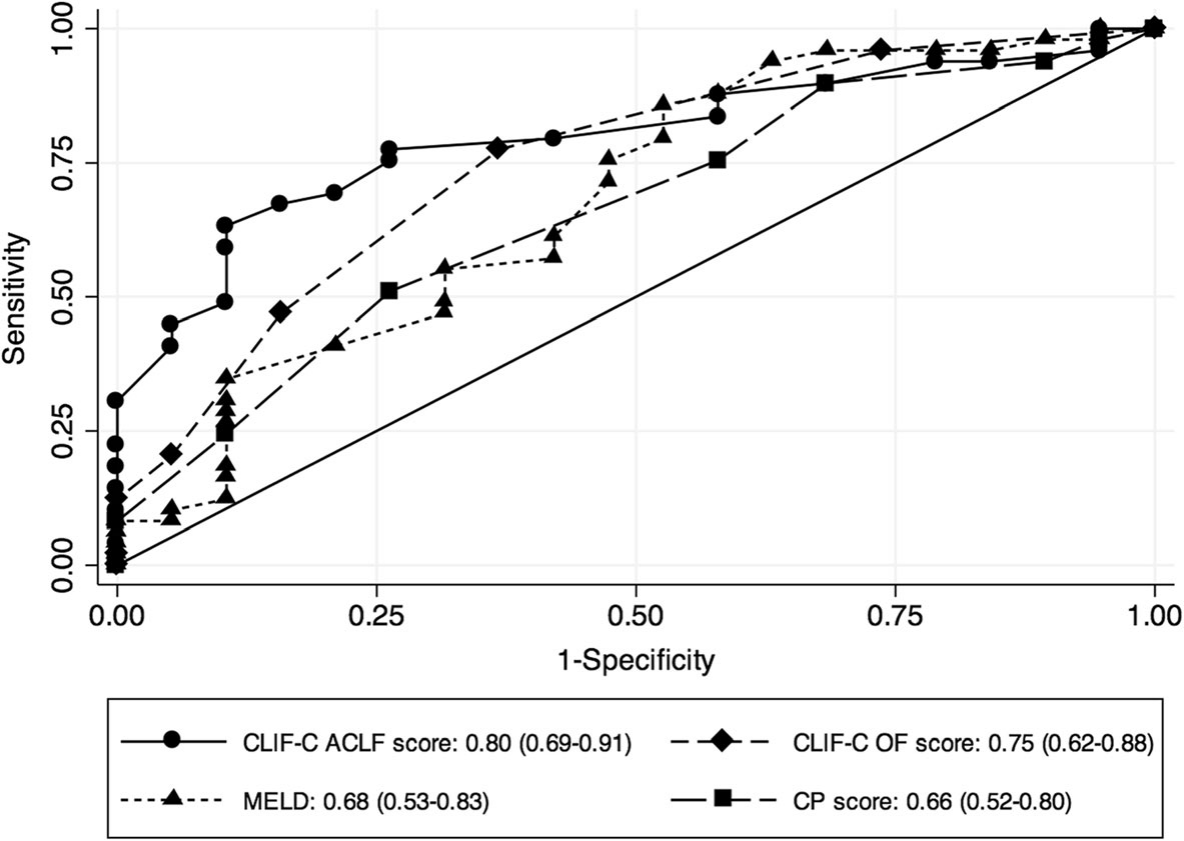
ROC curves of different prognostic scores in Acute-on-Chronic Liver Failure (ACLF) 3. The European Foundation for the study of chronic liver failure (CLIF-C) ACLF score had the best predictive value for 28-day mortality compared with all other scores. *CP* Child-Pugh, *MELD* Model for End-Stage Liver Disease, *OF* Organ failure

Because a previous study [5] has shown that patients with three or more organ failures incur high mortality, further analyses were conducted in patients with ACLF severity grade 3, which is defined by the presence of three or more organ failures [2]. The 28-day mortality in ACLF grade 3 was 72% (49 of 68), and none were transplanted. ROC analysis of all prognostic parameters that were significant upon univariate analysis revealed that the CLIF-C ACLF score had an AUROC of 0.80 (95% CI 0.69–0.91) for predicting 28-day mortality in ACLF grade 3 and was superior to MELD score, CLIF-C OF score, and Child-Pugh score (Fig. 2).

### Survival analysis in ACLF grade 3 according to CLIF-C ACLF score

**Fig. 3:**
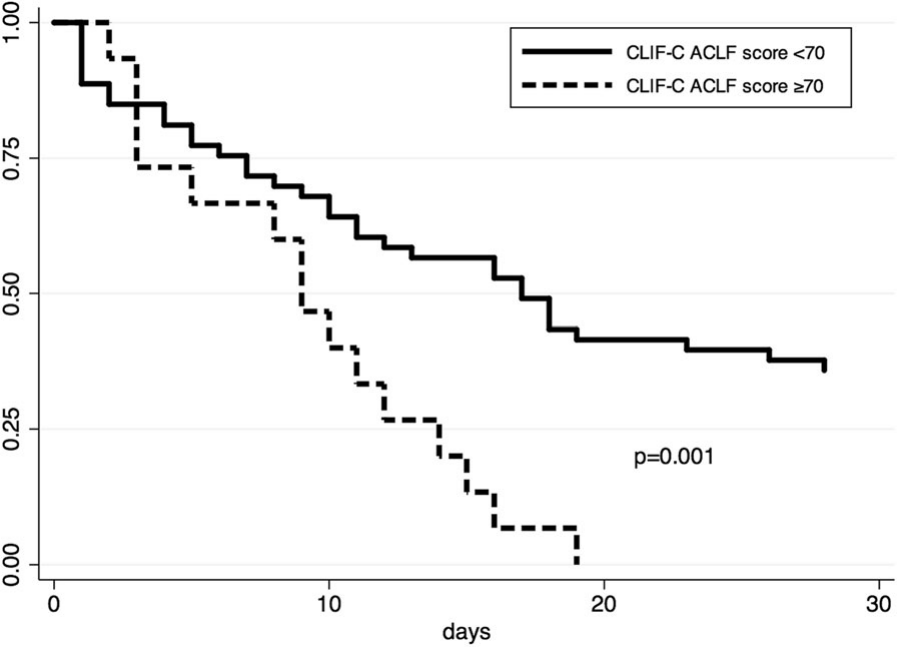
Twenty-eight-day survival according to the European Foundation for the study of chronic liver failure (CLIF-C) Acute-on-Chronic Liver Failure (ACLF) score in ACLF grade 3. Low 28-day survival is noted in patients with CLIF-C ACLF score ≥ 70, 2 days after receiving full intensive treatment unit supportive therapy

According to the ROC analysis, we depicted different thresholds for the CLIF-C ACLF score to assess their utility in predicting outcome in ACLF 3 patients (Table 3). Applying various thresholds for ACLF score in this population managed in the ICU, it is apparent that 28-day mortality varies from 80% in those with ACLF score ≥ 55 up to 100% in those with ACLF scores ≥ 70. Indeed, the highest specificity for determining 28-day mortality was seen with an ACLF score ≥ 70. Patients with a CLIF-C ACLF score below the threshold of 70 had a mortality of only 64% (34 of 53), which was significantly lower than in patients with ACLF score ≥ 70 (*p* = 0.006) (Fig. 3). The patients with ACLF grade 3 and ACLF score ≥ 70 were significantly older and had a higher number of organ failures. Parameters reflecting an inflammatory response (SIRS, WBC count) were also more elevated compared with patients below this cutoff (Table 4). Patients with CLIF-C ACLF score ≥ 70 incurred more renal failure (93.3% vs. 66%, *p* = 0.038; renal replacement 53% vs. 73%, *p* = 0.16) and a trend toward circulatory failure (87% vs. 62%; *p* = 0.07), whereas all other types of organ failure did not differ from CLIF-C ACLF score < 70 (Table 4).

**Table 3:** Mortality, sensitivity, and specificity for different thresholds of CLIF-C ACLF score

CLIF-C ACLF score	28-Day mortality	Sensitivity	Specificity
≥ 55	80% (95% CI 72–85)	88% (95% CI 75–95)	42% (95% CI 20–67)
≥ 60	88% (95% CI 78–94)	78% (95% CI 63–88)	74% (95% CI 49–91)
≥ 65	94% (95% CI 79–98)	59% (95% CI 44–73)	89% (95% CI 67–99)
≥ 70	100% (95% CI 78–100)	31% (95% CI 18–45)	100% (95% CI 82–100)

*Abbreviations: ACLF* Acute-on-Chronic Liver Failure, *CLIF* European Foundation for the study of chronic liver failure

**Table 4:** Clinical differences in ACLF 3 according to CLIF-C ACLF score

Parameters	CLIF-C ACLF score < 70 *n* = 53	CLIF-C ACLF score ≥ 70 *n* = 15	*p* Value
Age, years	50 ± 11	58 ± 9	*p* < 0.001
Male sex, *n* (%)	33 (62%)	10 (67%)	*p* = 0.76
Etiology of cirrhosis, *n* (%)			*p* = 0.52
ALD	28 (53%)	8 (53%)	
Autoimmune^a^	8 (15%)	1 (7%)	
Viral	5 (9%)	1 (7%)	
Viral + ALD	3 (6%)	1 (7%)	
NASH	1 (2%)	2 (13%)	
Cryptogenic + others	8 (15)	2 (13%)	
Precipitating event (infection/bleeding/both/unknown), *n* (%)	23/12/7/11 (43%/23%/13%/21%)	4/5/1/5 (27%/33%/7%/33%)	*p* = 0.46
CLIF-C OF score	14 (13–15)	16 (15–17)	*p* < 0.001
MELD	31.6 ± 8.7	38.1 ± 10.4	*p* = 0.02
SIRS score	2 (1–3)	3 (2–3)	*p* = 0.04
WBC (10^9^/L)	11 (6–17)	21 (15–24)	*p* = 0.002
Bilirubin, mg/dl; μmol/L	10.5 (4.3–15.8); 180 (74–270)	12.5 (6.9–22.8); 214 (118–390)	*p* = 0.43
Creatinine, mg/dl; μmol/L	1.7 (1.1–2.6); 150 (97–230)	2.4 (1.6–4.0); 212.2 (142–354)	*p* = 0.15
Sodium, mmol/L	135 ± 8	136 ± 15	*p* = 0.90
Renal replacement, *n* (%)	28 (53%)	11 (73%)	*p* = 0.16
Median number of organ failures	3 (3–3)	4 (3–5)	*p* < 0.001
Type of organ failure^a^			
Liver failure, *n* (%)	23 (43%)	9 (60%)	*p* = 0.255
Renal failure, *n* (%)	35 (66%)	14 (93%)	*p* = 0.038
Cerebral failure, *n* (%)	16 (30%)	5 (33%)	*p* = 0.816
Coagulation failure, *n* (%)	31 (58%)	11 (73%)	*p* = 0.296
Circulatory failure, *n* (%)	33 (62%)	13 (87%)	*p* = 0.074
Respiratory failure, *n* (%)	35 (66%)	9 (60%)	*p* = 0.666

*Abbreviations: ACLF* Acute-on-Chronic Liver Failure, *ALD* Alcoholic liver disease, *CLIF* European Foundation for the study of chronic liver failure, *MELD* Model for End-Stage Liver Disease, *NASH* Nonalcoholic steatohepatitis, *OF* Organ failure, *SIRS* Systemic inflammatory response syndrome, *WBC* White blood cell

Categorical variables are displayed in percent and continuous variables as mean ± SD (normally distributed data) and median (IQR) (nonparametric testing)

^a^Organ failures defined according to the CLIF-C OF score [2]

## Discussion

The data presented in this study suggest the CLIF-C ACLF score is the most accurate in predicting short-term (28-day) mortality for patients with ACLF compared with all other tested prognostic scores for chronic liver disease in patients with ACLF, especially for ACLF grade 3. We identified different thresholds of CLIF-C ACLF score to predict short-term mortality, and in order to maximize specificity around a threshold that would inform very high mortality and thereby question the benefit of ongoing ICU supportive care, further analyses were performed using a CLIF-C ACLF score cutoff ≥ 70. Applying a CLIF-C ACLF score cutoff ≥ 70 had 100% specificity for predicting mortality such that all patients above this threshold died within 28 days after ICU admission, despite maximal treatment efforts, including full organ support as per standard of care in our tertiary center. Maximal supportive treatment was provided up to the point that death was thought to be imminent. Despite the relatively limited number of patients with CLIF-C ACLF ≥ 70 (*n* = 15), our data suggest that ongoing intensive care support in these patients, in the absence of liver transplant, may be futile, given no improvement despite full organ support as needed for 48 hours. Our data are in line with previous reports showing a very poor prognosis in similar cohorts of at-risk patients, in whom dynamic assessments of change in CLIF-C ACLF score showed that those with further progression of ACLF grade or failed improvement had high mortality [5, 6]. The best management options in this scenario, given currently available limited therapies, require further evaluation, including the need for palliative care pathways.

The time point at which patients’ prognosis is assessed seems to be key. Our data suggest that mortality was relatively low (approximately 35–40%) within the first week after intensive treatment unit (ITU) admission, but beyond this, all remaining patients died quickly (within 2 weeks). This might imply that patients with CLIF-C ACLF ≥ 70 may have limited reserve and regenerative capacity, even if receiving full intervention support for the initial precipitating event. Moreover, the short survival period is an argument that either palliative care or, if eligible, liver transplant [10–12] should be discussed early after assessing the response to intensive care therapy for 48 hours, because the time until death and the window for intervention is very short thereafter.

Liver transplant in so-called high-MELD patients is highly debated because it is associated with significant posttransplant morbidity [13, 14]. Importantly, in our center, transplant selection aims at > 90% one-year survival, which necessitates the exclusion of urgently listing patients with decompensation or ACLF. However, there are data to suggest that overall survival can be in excess of 80% and comparable to patients transplanted without ACLF [12], which is also substantiated in other studies, including studies of living donor liver recipients [10, 15, 16]. By contrast, a retrospective study by Levesque et al. showed in a subgroup of 30 patients with ACLF grade 3 a 12-month survival rate of 43% after cadaveric liver transplant [11]. These studies clearly highlight that although it is worthwhile discussing liver transplant in ACLF grade 3, this must be tempered by assessment of factors that may indicate worse outcome after liver transplant, such as infections, age, and presence of hepatocellular carcinoma, as proposed by Levesque et al. [11]. In addition, patients through debilitation of their advanced liver disease and a continued severe inflammatory state, as seen with ACLF, would be expected to be frail and may not be rescued by liver transplant [17].

To date, interventions such as extracorporeal liver support, such as the trials with MARS (molecular adsorbent recirculation system) [18, 19], have failed to show any clear survival benefit in ACLF 3. When undertaking consideration for such interventions in such an advanced disease cohort, appropriate resource allocation and effectiveness of the intervention must remain major considerations for implementation. Until there are new interventions with proven efficacy, futility of ongoing intensive care support should be discussed early, also taking into consideration that cirrhosis and ACLF represent an increasing health and socioeconomic burden [20]. Such early decision-making processes help facilitate an appropriate and adequate palliative care option in a cohort in whom mortality is high, despite maximal intensive treatment support.

It is important to note that a CLIF-C ACLF score ≥ 70 was associated with distinct clinical features. Notably, the SIRS score and WBC count, which are reflective of an inflammatory response, were significantly higher in those with ACLF score ≥ 70, albeit that infections as specific precipitating events were not overrepresented and patients received antibiotic treatment as part of the standard procedure. This in line with the assertion that increasing disease severity in ACLF is accompanied by a systemic inflammatory response. Claria et al. [21] and others have shown that proinflammatory cytokines increase throughout the different severity grades of ACLF and that such inflammation is associated with higher mortality [22–24]. This may imply that strategies to lower inflammation and thereby risk of new infection, such as gut decontamination, may improve outcomes, but further clinical trials of such interventions are needed. Moreover, it remains to be seen whether these strategies are cost-effective in such sick patients.

There are some limitations of this study that need consideration. First, this study is a retrospective analysis of prospectively gathered data, which may be regarded as a weakness because some potential contributory factors that might influence outcome may not have been assessed at the time of enrollment. Second, a further potential limitation of this study is that the response to supportive therapy in the ICU was evaluated at 48 hours and not beyond. The previously reported outcomes in ACLF grade 3 patients in the CANONIC study by Gustot et al. showed that assessment of CLIF-C ACLF score between days 3 and 7 and a change in score determined longer-term outcome. This supports the idea of repeated assessments to define futility in such patients, in whom a fixed time of assessment may sometimes be difficult [5].

## Conclusions

Patients with ACLF who require intensive care supportive treatment should be assessed early after ITU admission using the CLIF-C ACLF score. In patients with ACLF 3 and a CLIF-C ACLF score ≥ 70, who are not suitable for liver transplant, futility of continued currently available intensive supportive therapy should be considered. The best treatment options in this scenario remain to be determined but may include palliative care.

## Additional file

Additional file 1: **Table S1.** All collected parameters were analyzed using univariate Cox regression to identify potential predictors of 28-day mortality. (DOCX 17 kb)

## Abbreviations

ACLF: Acute-on-chronic liver failure
AUROC: Area under the receiver operator characteristic
CLIF: European Foundation for the study of chronic liver failure
CLIF-OF: European Foundation for the study of chronic liver failure organ failure score
CP: Child-Pugh
ICU: Intensive care unit
INR: International normalized ratio
ITU: Intensive treatment unit
MELD: Model for End-Stage Liver Disease
NASH: Nonalcoholic steatohepatitis
SIRS: Systemic inflammatory response syndrome
SOFA: Sequential Organ Failure Assessment
WBC: White blood cell

## References

[1] Moreau R, Jalan R, Gines P, Pavesi M, Angeli P, Cordoba J (2013). Acute-on-chronic liver failure is a distinct syndrome that develops in patients with acute decompensation of cirrhosis. Gastroenterology.

[2] Jalan R, Saliba F, Pavesi M, Amoros A, Moreau R, Gines P (2014). Development and validation of a prognostic score to predict mortality in patients with acute-on-chronic liver failure. J Hepatol.

[3] Olson JC, Wendon JA, Kramer DJ, Arroyo V, Jalan R, Garcia-Tsao G (2011). Intensive care of the patients with cirrhosis. Hepatology.

[4] Jalan R, Gines P, Olson JC, Mookerjee RP, Moreau R, Garcia-Tsao G (2012). Acute-on-chronic liver failure. J Hepatol.

[5] Gustot T, Fernandez J, Garcia E, Morando F, Caraceni P, Alessandria C (2015). Clinical course of acute-on-chronic liver failure syndrome and effects on prognosis. Hepatology.

[6] Cardoso FS, Pereira R, Alexandrino G, Bagulho L (2017). Futility of care in patients with acute-on-chronic liver failure. Hepatology.

[7] Jalan R, Stadlbauer V, Sen S, Cheshire L, Chang YM, Mookerjee RP (2012). Role of predisposition, injury, response and organ failure in the prognosis of patients with acute-on-chronic liver failure: a prospective cohort study. Crit Care.

[8] Sawhney R, Holland-Fischer P, Rosselli M, Mookerjee RP, Argawal B, Jalan R (2016). Role of ammonia, inflammation, and cerebral oxygenation in brain dysfunction of acute-on-chronic liver failure patients. Liver Transpl.

[9] D’Amico G, Garcia-Tsao G, Pagliaro L (2006). Natural history and prognostic indicators of survival in cirrhosis: a systematic review of 118 studies. J Hepatol.

[10] Moon DB, Lee SG, Kang WH, Song GW, Jung DH, Park GC (2017). Adult living donor liver transplantation for acute-on-chronic liver failure in high-model for end-stage liver disease score patients. Am J Transplant.

[11] Levesque E, Winter A, Noorah Z, Daures JP, Landais P, Feray C (2017). Impact of acute-on-chronic liver failure on 90-day mortality following a first liver transplantation. Liver Int.

[12] Artru F, Louvet A, Ruiz I, Levesque E, Labreuche J, Ursic-Bedoya J (2017). Liver transplantation in the most severely ill cirrhotic patients: a multicentre study in acute-on-chronic liver failure grade 3. J Hepatol.

[13] Xia VW, Du B, Braunfeld M, Neelekanta G, Hu KQ, Nourmand H (2006). Preoperative characteristics and intraoperative transfusion and vasopressor requirements in patients with low vs. high MELD scores. Liver Transplant.

[14] Marubashi S, Dono K, Asaoka T, Hama N, Gotoh K, Miyamoto A (2006). Risk factors for graft dysfunction after adult-to-adult living donor liver transplant. Transplant Proc.

[15] Bernal W, Jalan R, Quaglia A, Simpson K, Wendon J, Burroughs A (2015). Acute-on-chronic liver failure. Lancet.

[16] Jalan R, Yurdayclin C, Bajaj JS, Acharya SK, Arroyo V, Lin HC (2014). Toward an improved definition of acute-on-chronic liver failure. Gastroenterology.

[17] Underwood PW, Cron DC, Terjimanian MN, Wang SC, Englesbe MJ, Waits SA (2015). Sarcopenia and failure to rescue following liver transplantation. Clin Transpl.

[18] Banares R, Nevens F, Larsen FS, Jalan R, Albillos A, Dollinger M (2013). Extracorporal albumin dialysis with the molecular adsorbent recirculating system in acute-on-chronic liver failure: the RELIEF trial. Hepatology.

[19] Hessel FP, Bramlage P, Wasem J, Mitzner SR (2010). Cost-effectiveness of the artificial liver support system MARS in patients with acute-on-chronic liver failure. Eur J Gastroenterol Hepatol.

[20] Allen AM, Kim WR, Moriarty JP, Shah ND, Larson JJ, Kamath PS (2016). Time trends in the health care burden and mortality of acute-on-chronic liver failure in the United States. Hepatology.

[21] Claria J, Stauber RE, Coenraad MJ, Moreau R, Jalan R, Pavesi M (2016). Systemic inflammation in decompensated cirrhosis: characterization and role in acute-on-chronic liver failure. Hepatology.

[22] Wu W, Yan H, Zhao H, Sun W, Yang Q, Sheng J (2018). Characteristics of systemic inflammation in hepatitis B-precipitated ACLF: differentiate it from no-ACLF. Liver Int.

[23] Alcaraz-Quiles J, Titos E, Casulleras M, Pavesi M, Lopez-Vicario C, Rius B (2017). Polymorphisms in the IL-1 gene cluster influence systemic inflammation in patients at risk for acute-on-chronic liver failure. Hepatology.

[24] Arroyo V, Moreau R, Jalan R, Ginès P, EASL-CLIF Consortium CANONIC Study (2015). Acute-on-chronic liver failure: a new syndrome that will re-classify cirrhosis. J Hepatol.

[25] Vilstrup Hendrik, Amodio Piero, Bajaj Jasmohan, Cordoba Juan, Ferenci Peter, Mullen Kevin D., Weissenborn Karin, Wong Philip (2014). Hepatic encephalopathy in chronic liver disease: 2014 Practice Guideline by the American Association for the Study Of Liver Diseases and the European Association for the Study of the Liver. Hepatology.

