# Supplementary material for: Validation of CLIF-C ACLF score to define a threshold for futility of intensive care support for patients with acute-on-chronic liver failure
Source: Crit Care. 2018 Oct 10;22:254. doi: 10.1186/s13054-018-2156-0 (PMC6180662; doi:10.1186/s13054-018-2156-0)
Supplement: Supplementary file 1 — Table S1. All collected parameters were analyzed using univariate Cox regression to identify potential predictors of 28-day mortality. (DOCX 17 kb) [file 13054_2018_2156_MOESM1_ESM.docx]

Additional file 1

**Table S1.** All collected parameters were analysis in a univariate Cox-regression analysis in order to identify potential predictors for 28-day mortality.

|  | Univariate analysis | |
| --- | --- | --- |
| Parameter | Hazard ratio | p value |
| MELD score | 1.05 (95%CI 1.03-1.07) | p<0.0001 |
| Child-Pugh score | 1.36 (95%CI 1.21-1.54) | p<0.0001 |
| CLIF-C ACLF score | 1.07 (95%CI 1.04-1.09) | p<0.0001 |
| Gender | 1.06 (0.70-1.60) | 0.79 |
| Age (years) | 1.01 (1.00-1.03) | 0.15 |
| Sodium (mmol/L) | 0.99 (0.97-1.01) | 0.22 |
| Albumin (g/L) | 0.98 (0.95-1.01) | 0.14 |
| Platelet count (10^9^/L) | 1.00 (0.99-1.00) | 0.01 |
| Ascites | 1.25 (0.83-1.89) | 0.29 |
| HE (0-2/3-4)^+^ | 1.63 (1.00-2.63) | 0.048 |
| Bilirubin (µmol/L) | 1.01 (1.00-1.03) | 0.05 |
| INR | 1.36 (1.20-1.54) | <0.0001 |
| Creatinine (µmol/L) | 1.00 (0.94-1.07) | 0.98 |
| WBC (10^9^/L)^a^ | 1.00 (0.98-1.03) | 0.95 |

- MELD – model of endstage liver disease
- CLIF – chronic liver failure
- OF – organ failure
- ACLF – acute-on-chronic liver failure
- ^+^ HE – hepatic encephalopathy; classification according to West-Haven-Criteria [18]
- ^a^ WBC count – white blood cell count
